# Reintegration into school, kindergarten and work in families of childhood cancer survivors after a family-oriented rehabilitation program

**DOI:** 10.3389/fped.2024.1288567

**Published:** 2024-03-07

**Authors:** Laura Inhestern, Mona L. Nasse, Konstantin A. Krauth, Daniela Kandels, Stefan Rutkowski, Gabriele Escherich, Corinna Bergelt

**Affiliations:** ^1^ Department of Medical Psychology, University Medical Center Hamburg-Eppendorf, Hamburg, Germany; ^2^ Department of Pediatrics, Pediatric Hematology & Oncology, Klinik Bad Oexen, Bad Oeynhausen, Germany; ^3^ Swabian Children’s Cancer Center, University Hospital Augsburg, Augsburg, Germany; ^4^ Department of Pediatric Hematology and Oncology, University Medical Center Hamburg-Eppendorf, Hamburg, Germany; ^5^ Department of Medical Psychology, University Medicine Greifswald, Greifswald, Germany

**Keywords:** pediatric cancer, rehabilitation, quality of life, chronic (health) condition, reintegration

## Abstract

**Objective:**

To describe the situation of childhood cancer survivors and their parents before and one year after a family-oriented rehabilitation program (FOR) and to identify factors influencing reintegration.

**Methods:**

We included parents of children diagnosed with leukemia or central nervous system tumor. We assessed parental functioning using the functioning subscale of the Ulm Quality of Life Inventory for Parents (ULQIE) and children's school/kindergarten related quality of life (parental assessment, subscale KINDL-R). Descriptive analyses, group comparisons and multiple regression analyses on data of 285 parents of 174 children diagnosed with leukemia or central nervous system tumor.

**Results:**

Parents reported changes in their work situation (e.g., reduction of working hours) due to their child's diagnosis. Parental functioning increased significantly over time. Children's leukemia diagnosis and shorter time since the end of treatment were associated with higher functioning in parents one year after FOR. Parents reported difficulties in the child's work pace, concentration, stress resilience and empathy. The school/kindergarten-related quality of life (QoL) of the children was lower than in the general population. One year after FOR, most children reintegrated fully in school/kindergarten, partly with support (e.g., integration assistant). No significant predictors for children's reintegration were identified.

**Discussion:**

Parents and children experience major changes in their work/school/kindergarten life. One year after FOR most parents reported a reintegration of their children, however the children's school/kindergarten-related QoL remained below average compared to norm values. Even after rehabilitation families of childhood cancer survivors might benefit from psychosocial and practical support offers to support families with the reintegration into work/school/kindergarten.

## 1 Introduction

Improved diagnostic methods and treatment strategies lead to a growing population of childhood cancer survivors in the developed countries (1). Nowadays, the 5-year survival probability after pediatric cancer is approximately 80% (1). Even though most survivors and parents adapt well after the end of intensive cancer treatment (2–6), some affected persons report high psychosocial stress levels even years after the end of treatment (5–12).

Apart from the psychosocial burden, survivors and parents can experience long-term changes in their school or kindergarten situation and in their work life. Under treatment, many survivors are unable to attend school regularly due to their illness, low immunity or hospital stays (13). Some survivors also feel isolated when returning to school and suffer from negative reactions of their classmates (13). After treatment, some children and particularly survivors of cancers of the central nervous system (CNS) still report educational or social problems in school (14). Follow-up programs that support childhood cancer survivors with their reintegration into school and with social skills are therefore indicated (14).

The work situation of parents of childhood cancer patients is also highly impacted by the diagnosis, the treatment and long-term effects. Some parents, in particular mothers, take sick leave, reduce their working hours or terminate their employment after their child's diagnosis to look after the ill child and the patient's siblings (15–18). Some parents also report job loss, financial problems and reduced functioning at work (15–18). Adverse changes in the employment situation and income of parents last beyond the end of treatments in some families (19–21).

In Germany, a 4-week inpatient family-oriented, multicomponent rehabilitation program (FOR) is implemented to ensure the treatment success in the long term, but also to follow individual rehabilitation goals of all family members after cancer treatment (22, 23). FOR is covered by the health or pension insurance. The treatment plan within the rehabilitation is individualized based on the physical and emotional situation of the patient/family member (24). The FOR concept follows the World Health Organization's (WHO) holistic understanding of impairments and functional health described in the International Classification of Functioning, Disability and Health (ICF) (25). Thus, FOR's overarching aim is to support the child's participation and reintegration into kindergarten and school as well as the parents' functioning and reintegration to work.

The reintegration into school/kindergarten or work of childhood cancer survivors or their parents after a FOR has not yet been investigated. Therefore, this study aims to describe the school/kindergarten situation of childhood cancer survivors and the work situation of their parents at the beginning of and one year after the end of FOR and to identify predictors for the reintegration into school/kindergarten and work.

## 2 Methods

### 2.1 Design

The results of this study are part of a prospective observational study with a longitudinal mixed-methods design (26). Findings of the study on quality of life, fear of recurrence and qualitative interviews have been published elsewhere (27–32). The overall study was approved by the Ethics Committee of the Medical Chamber of Hamburg (number: PV5277). The reporting of this article follows the Statistical Analyses and Methods in the Published Literature (SAMPL) guideline (33).

### 2.2 Participants and procedure

In this study, we focused on the most frequent childhood cancer diagnoses in Germany, central nervous system (CNS) tumors and leukemia (34). We surveyed parents (biological parents and other caregivers) whose children had been diagnosed with CNS-tumors or leukemia under the age of 18 years, who had completed the intensive cancer treatment and participated in a FOR. We excluded parents with high physical or mental burden (applicable if the study participation would be overly burdensome), cognitive limitations, insufficient German language skills to answer the questionnaires and parents who refused to participate in the study. The inclusion and exclusion criteria were assessed by rehabilitation physicians in the cooperating rehabilitation clinic.

The physicians in the clinic informed the parents about the study at the beginning of the inpatient rehabilitation program and gave out written study information, consent forms for participation and the questionnaires for the first measurement time point (beginning of FOR). Additionally, the physicians documented basic medical information on the patient. One year after the end of FOR, the parents received the final questionnaires via postal mail by the rehabilitation clinic. A detailed description of the recruitment process is presented in the study protocol (26).

### 2.3 Measures

#### 2.3.1 Sociodemographic and medical data

Sociodemographic data of the childhood cancer survivors and their parents were assessed via the parent's questionnaire at the beginning of FOR. At the first measurement time point, parents were asked about changes in their work situation (reduction or increase of working time, other changes) since the diagnosis. One year after the end of FOR, they were asked about changes since the end of FOR. Medical data were either extracted from the physician's questionnaire. If not reported in the physicians questionnaire, we used medical information given in the parent's questionnaire at the beginning of FOR. The children's physical functioning at the beginning and end of the rehabilitation program was assessed by the physicians in the clinic in steps of ten percent ranging from 0%–100%.

#### 2.3.2 Parental functioning

Since the professional situation and reintegration process of parents is very complex, we decided to measure the parents' functioning at the beginning of the FOR and one year after the end of FOR as a basic requirement for professional reintegration. Therefore, we assessed parental functioning with the 7-item functioning subscale of The Ulm Quality of Life Inventory for Parents (ULQIE) (35). The items (e.g., In the last week, I was fully efficient at work/housekeeping) focus on the past week and are scored on a 5-point Likert scale from never (0) to always (4). The functioning subscale has adequate psychometric properties (28).

#### 2.3.3 Parental anxiety

We measured parental anxiety at the beginning of FOR using the 7-item Generalized Anxiety Disorder Screener (GAD-7) (36). Participants report on a 4-point Likert scale from not at all (0) to nearly every day (3) how often they have felt burdened by anxiety symptoms as described in the Diagnostic and Statistical Manual of Mental Disorders (DSM-IV) (37, 38). The GAD-7 is a reliable and valid measure of anxiety (38).

#### 2.3.4 Parental depression

Depressive symptoms in parents was measured with the 9-item depression module (PHQ-9) of the Patient Health Questionnaire (PHQ) at the beginning of FOR (39). The items reflect depression symptoms as described in the DSM-IV (37). Answers are given on a 4-point Likert scale from not at all (0) to nearly every day (3). The PHQ-9 has adequate psychometric properties (40, 41).

#### 2.3.5 Children's school/kindergarten related quality of life and skills

The survivors school/kindergarten related quality of life at the beginning of and one year after the end of FOR was measured with the 4-item subscale school or nursery school/kindergarten of the Questionnaire for Measuring Health-Related Quality of Life in Children and Adolescents (KINDL-R) (42, 43). Parents rate their child's school/kindergarten related quality of life of the past seven days on a 5-point Likert scale from never (1) to all the time (5). Raw values are transformed to a 0–100 scale for comparisons with norm values from the general population. The KINDL-R is a reliable and valid measure (44, 45).

Additionally, we measured ICF related skills that are relevant for a successful reintegration into school or kindergarten via eight self-developed items at the beginning of the FOR and one year after the end of FOR (Table 3). Parents rated on a scale from never (1) to always (5) how well their ill child masters different tasks (e.g., performing actions in an adequate work pace, understanding feelings and thoughts of others) according to his or her age.

### 2.4 Statistical analyses

We conducted descriptive analyses to describe sociodemographic and medical characteristics as well as the school/kindergarten situation of children and the work situation of their parents. Differences between mothers and fathers and between measurement points were calculated using Chi^2^-tests for categorical variables and *t*-tests for continuous variables (two-sample *t*-tests for differences between mothers and fathers, paired *t*-tests for differences between measurement points). In order to examine a potential selection bias, we used Pearson Chi^2^-tests to detect potential differences between the ICF ratings of parents that participated only at the beginning of the FOR and the ratings of parents that participated at both measurement time points.

Multiple regression analyses (method: enter) were used to identify predictors of parental functioning and the children's school/kindergarten related quality of life one year after the end of FOR. The independent variables were assessed at the beginning of FOR. In the regression model on parental functioning one year after the end of FOR (dependent variable), we controlled for the functioning at the beginning of the FOR and the time since first measurement time point. The following independent variables were included in the regression model: gender, education, parental depression, parental anxiety, child's diagnosis, child's age, time since end of treatment, number of child's siblings. In the regression model of the child's school/kindergarten related quality of life one year after the end of FOR (dependent variable), we controlled for the variables school/kindergarten related quality of life at the beginning of FOR, time since first measurement time point and the gender of the rater. We included the following independent variables: age, gender, diagnosis, physical functioning, time since end of treatment, number of siblings and parental functioning.

All analyses were performed with the software IBM SPSS Statistics 27. Missing values in the validated measures were imputed with the individual mean with a maximum of 30% missing data within one scale. Alpha was set at *p *< .05 (two-sided) for all analyses.

## 3 Results

### 3.1 Sample characteristics

From July 2016 to December 2018 the cooperating rehabilitation clinic identified 237 families that were potentially eligible for study participation. 177 families answered the questionnaires. 60 families did not participate for the following reasons: physical or mental burden (self-assessment, *n *= 12), cognitive limitations (*n *= 3), insufficient German language skills (*n *= 14), refusal of participation (*n *= 21), not specified (*n *= 10). Two families were excluded subsequently due to a diagnosis other than leukemia or CNS tumor. In one family only children answered questionnaires. We analyzed the data of 285 parents of 174 families at the beginning of FOR and of 149 parents of 90 families one year after end of rehabilitation. At the first measurement time point, the mean age of the 285 parents (60% mothers) was *M *= 39.2 (*SD *= 7.3, Table 1). The patient's mean age was *M *= 7.3 (*SD *= 4.0). 64% had a leukemia diagnosis and 36% a CNS tumor diagnosis and the mean time since diagnosis was *M *= 24.9 months (*SD *= 25.8).

**Table 1:** Sociodemographic data of 285 parents of 174 childhood cancer survivors.

Sociodemographic data							*p* ^a^
Parents	Total (*n *= 285)		Fathers (*n *= 115)		Mothers (*n *= 170)		
	*M*	*SD*	*M*	*SD*	*M*	*SD*	* *
Age in years	39.2	7.3	40.6	7.4	38.2	7.0	.119
	*n*	%	*n*	%	*n*	%	
Permanent relationship	263	92.3	113	98.3	150	88.2	.002
Number of children^b^							
0	1	0.4	1	0.9	-	-	-^k^
1	65	22.9	21	18.4	44	25.9	
2–3	201	70.8	85	74.5	116	68.2	
>3	17	6.0	7	6.1	10	5.9	
Education^c^							
>10 years	136	50.4	56	51.9	80	49.4	.748
≤10 years	134	49.6	52	48.1	82	50.6	
Main income earner^d^	121	46.6	92	86.8	29	18.8	<.001
Employment status^e^							
Gainfully employed	201	72.3	105	92.9	96	58.2	-^k^
Full-time	118	58.7	98	93.3	20	20.8	
Part-time	83	41.3	7	6.7	76	79.2	
Not gainfully employed	57	20.5	5	4.4	52	31.5	
Homemakers	32	56.1	-	-	32	61.5	
(re)training	2	3.5	-	-	2	3.8	
Seeking employment	22	38.6	4	80.0	18	34.6	
Retired	1	1.8	1	20.0	-	-	
Other, e.g., parental leave	20	7.2	3	2.7	17	10.3	
Current sick leave^e^	48	17.3	16	14.2	32	19.4	.257
Duration of sick leave in months (*M*, *SD*/range)^f^	10.9	5.8	8.0	6.4	12.1	5.4	.096
Monthly net household income^g^							
<1,250€	15	5.6	3	2.8	12	7.6	.028
1,250€–1,749€	23	8.6	3	2.8	20	12.7	
1,750€–2,249€	28	10.5	11	10.1	17	10.8	
2,250€–2,999€	53	19.9	20	18.3	33	20.9	
3,000€–3,999€	74	27.7	37	33.9	37	23.4	
4,000€–4,999€	33	12.4	16	14.7	17	10.8	
≥5,000€	41	15.4	19	17.4	22	13.9	

Patients	Total (*n *= 174)		Boys (*n *= 95)		Girls (*n *= 79)		
	*M*	*SD*	*M*	*SD*	*M*	*SD*	* *
Age in years	7.3	4.0	7.9	4.2	6.5	3.6	.017
Time since diagnosis in months	24.9	25.8	25.9	25.8	23.7	25.8	.549
	*n*	%	*n*	%	*n*	%	
Number of siblings							
0	38	21.8	19	20.0	19	24.1	.441
1–2	125	71.8	68	71.6	57	72.2	
>2	11	6.3	8	8.4	3	3.8	
Cancer diagnosis							
CNS tumor	62	35.6	35	36.8	27	34.2	.715
Leukemia	112	64.4	60	63.2	52	65.8	
Other chronic diseases or impairments, e.g., epilepsy, hemiparesis	42	24.1	21	22.1	21	26.6	.655
Kindergarten (or similar institution) currently or in the past^h^							
Yes	140	83.3	81	88.0	59	77.6	.200
Yes, similar institution	8	4.8	2	2.2	6	7.9	
No	11	6.5	5	5.4	6	7.9	
No, too young	9	5.4	4	4.3	5	6.6	
School enrolment^i^							
Timely	75	47.5	47	54.0	28	39.4	.203
Premature	3	1.9	1	1.1	2	2.8	
Deferred	18	11.4	12	13.8	6	8.5	
Too young for enrolment	62	39.2	27	31.0	35	49.3	
School career^j^							
Regular	57	37.7	34	41.5	23	33.3	-^k^
Class repeated once	16	10.6	11	13.4	5	7.2	
Multiple classes repeated	1	0.7	1	1.2	-	-	
Other, e.g., home schooling, special needs school)	10	6.6	6	7.3	4	5.8	
Not going to school	67	44.4	30	36.6	37	53.6	

^a^ Chi^2^-Test or *t*-test.

^b^ One missing.

^c^ 15 missings.

^d^ 25 missings.

^e^ 7 missings.

^f^ 19 missings.

^g^ 18 missings.

^h^ 6 missings.

^i^ 16 missings.

^j^ 23 missings.

^k^ No significance test due to small number in cells.

### 3.2 Work situation and functioning of parents

At the beginning of FOR, 72% of the parents were gainfully employed (Table 1). Employment status significantly differed between mothers and fathers (*p* < .001). Whereas 93% of the fathers were working full-time, 21% of the mothers did. 56% of the parents reported a monthly net household income of 3,000€ and above. 17% of the parents were on sick leave at the first measurement time point. The mean duration of sick leave was *M *= 10.9 months (*SD *= 5.8).

At the beginning of the FOR, parents were asked to report changes in their work situation from the time of diagnosis until the time of the survey. 17% of the parents reported a reduction of their working hours, 2% an increase of their working hours and 39% reported other changes (Table 2). Other reported changes at the beginning of the FOR included, inter alia, sick leave, unpaid leave, termination, time for nursing care, parental leave, job change and an increased use of work from home. Changes were mainly reported by mothers (*p* < .001). One year after the end of FOR, parents reported changes in their work life since the end of FOR. 9% of the parents reported a reduction of working hours, 10% an increase of their working hours and 19% reported other changes. Again, changes were more prevalent in mothers (*p* = .001). The parents mean functioning measured with the functioning subscale of the ULQIE was *M *= 15.5 (*SD *= 5.2) at the beginning of the FOR and *M *= 17.9 (*SD *= 5.6) one year after the end of FOR. There was a significant increase in parents' functioning over time (*p *< .001). While fathers reported a significantly higher functioning than mothers at the beginning of FOR (*p *= .013), there was no significant difference in functioning one year later (*p *= .179).

**Table 2:** Changes in parents’ professional situation and functioning (ULQIE) reported at the beginning of the FOR and one year later (multiple responses possible).

Changes in professional situation and functioning	Beginning of the FOR			One year after end of FOR		
	Total	Fathers	Mothers	Total	Fathers	Mothers
	(*n *= 285)	(*n *= 115)	(*n *= 170)	(*n *= 149)	(*n *= 59)	(*n *= 90)
	*n* (%)	*n* (%)	*n* (%)	*n* (%)	*n* (%)	*n* (%)
Reduction of working time	48 (16.8)	14 (12.2)	34 (20.0)	13 (8.7)	3 (5.1)	10 (11.1)
Increase of working time	5 (1.8)	2 (1.7)	3 (1.8)	15 (10.1)	5 (8.5)	10 (11.1)
Other changes	110 (38.6)	38 (33.0)	72 (42.4)	28 (18.8)	8 (13.6)	20 (22.2)
	*M (SD)*	*M (SD)*	*M (SD)*	*M (SD)*	*M (SD)*	*M (SD)*
Functioning (ULQIE)^a^	15.5 (5.2)	16.5 (5.2)	14.9 (5.1)	17.9 (5.6)	18.6 (5.9)	17.4 (5.4)

^a^ Beginning of FOR 3 missings, end of FOR 1 missing, possible range 0–28.

### 3.3 School/kindergarten situation of childhood cancer survivors

At the beginning of FOR, parents reported that 83% of the children went to a kindergarten or a similar institution at the time of the survey or in the past (Table 1). The school enrollment of 11% of the children was deferred. The school career of most survivors was regular, but 11% of the survivors had had to repeat a class once or multiple times. ICF related skills that are relevant for a successful reintegration into kindergarten or school were assessed by the parents at the beginning of the FOR and one year later (Table 3). There were no significant differences between the ratings of parents that participated only at the beginning of the FOR and the ratings of parents that participated at both measurement time points (see Supplementary Table S1).

**Table 3:** Changes in survivors’ ICF related skills and their health-related quality of life with regard to school/kindergarten assessed by the parents at the beginning of the FOR and one year later.

	Beginning of the FOR			One year after the end of FOR		
	Total (*n *= 174)	Boys (*n *= 95)	Girls (*n *= 79)	Total (*n *= 90)	Boys (*n *= 50)	Girls (*n *= 40)
“Please specify how well your child masters the following tasks according to his or her age.”^a,b^	*M (SD)*	*M (SD)*	*M (SD)*	*M (SD)*	*M (SD)*	*M (SD)*
Solving tasks that require capacity of memory	3.8 (1.0)	3.8 (1.0)	3.7 (0.9)	3.9 (1.0)	3.9 (1.0)	3.8 (1.0)
Performing actions in an adequate work pace	3.4 (0.9)	3.4 (0.9)	3.4 (0.8)	3.4 (1.0)	3.4 (0.9)	3.3 (1.0)
Concentrating	3.5 (0.9)	3.5 (0.9)	3.4 (0.9)	3.5 (0.9)	3.5 (0.9)	3.4 (0.9)
Having energy for school/kindergarten	3.8 (0.9)	3.8 (1.0)	3.8 (0.8)	3.8 (0.9)	3.8 (1.0)	4.0 (0.9)
Understanding and solving tasks	3.7 (0.9)	3.7 (0.8)	3.8 (0.9)	3.9 (0.8)	4.0 (0.9)	3.8 (0.8)
Listening or observing attentively	3.8 (0.9)	3.8 (0.9)	3.8 (1.0)	3.9 (0.9)	3.9 (1.0)	3.9 (0.8)
Withstanding stress	3.0 (1.0)	3.0 (0.9)	2.9 (0.9)	3.1 (0.9)	3.0 (0.9)	3.1 (0.9)
Understanding feelings and thoughts of others	3.6 (1.0)	3.5 (1.0)	3.7 (1.0)	3.8 (0.9)	3.7 (0.9)	3.9 (1.0)
	*M (SD)*	*M (SD)*	*M (SD)*	*M (SD)*	*M (SD)*	*M (SD)*
KINDL-R subscale school^c^	73.9 (16.8)	72.6 (17.6)	75.8 (15.6)	72.4 (18.9)	72.0 (19.8)	72.9 (17.8)

^a^ Scale: 1 = never, 5 = always.

^b^ 5–14 missings at the beginning of FOR, 1 missing each at the end of FOR.

^c^ 46 missings at the beginning of FOR, 8 missings at the end of FOR, 0–100 transformed scale.

Difficulties were particularly reported with regard to the survivors' child's work pace, concentration, stress resilience and, after the end of FOR, additionally with regard to empathy. The health-related quality of life with regard to school/kindergarten was *M *= 73.9 (*SD *= 16.8) at the beginning and *M *= 72.4 (*SD *= 18.9) one year after the end of FOR. At both measurement time points, there were no significant gender differences in the KINDL-R school subscale score (beginning of FOR: *p *= .285; one year after end of for: *p *= .831). Further, the KINDL-R school subscale score did not change significantly over time (*p *= .398). Exploratory comparisons of patients with CNS and leukemia, significant differences were observed for some ICF related skills at the beginning of the FOR but not one year after FOR (see Supplementary Tables S2–S4).

One year after the end of FOR, parents were asked to describe the reintegration into school or kindergarten of their child. At this measurement time point, four children had returned to school/kindergarten on an hourly basis, 22 children had returned hourly until the full reintegration, 57 had reintegrated fully after the end of intensive cancer treatment and for 7 children, the parents did not provide any information on the reintegration process. Eighteen survivors were supported by an integration assistant and 13 children received other support (e.g., transport service, school support assistant, compensation of disadvantages at school).

### 3.4 Predictors of parental functioning and school/kindergarten related quality of life

The regression model on parents' functioning one year after the end of FOR (dependent variable) accounted for 38% of the variance (adjusted *R^2 ^*= .38, *p *< .01, *n *= 136, Table 4). Parents of CNS tumor survivors reported significantly worse functioning than parents of leukemia survivors (*β* = .143, *p *= .047). Moreover, time since end of treatment was associated with functioning in parents (*β* = −.148, *p *= .045).

**Table 4:** Predictors of parent's functioning one year after the end of FOR (*n *= 136)^a^.

	*B*	*SE*	Beta	*T*	*p*
Functioning at the beginning of FOR	0.325	0.098	.312	3.300	.001
Time since first measurement time point	−0.012	0.351	-.002	−0.034	.973
Gender (reference: female)	0.105	0.841	.009	0.125	.901
Education (reference: >10 years)	0.158	0.767	.014	0.206	.837
Depression	−0.217	0.125	−.205	−1.736	.085
Anxiety	−0.224	0.124	−.191	−1.809	.073
Child’s diagnosis (reference: leukemia)	1.649	0.822	.143	2.006	.047
Child’s age	0.127	0.107	.084	1.189	.237
Time since end of treatment	−0.050	0.025	−.148	−2.022	.045
Number of survivor’s siblings	0.441	0.437	.071	1.010	.314
*R*^2^ (adjusted *R*^2^)	.43 (.38)				
*F*	9.308				
*P*	<.001				

^a^ Controlled for the time since first measurement time point.

The regression model on child's school/kindergarten related quality of life one year after the end of FOR (dependent variable) accounted for 16% of the variance (adjusted *R^2 ^*= .16, *p *= .035, *n *= 62), Table 5. Besides the control variable school/kindergarten related quality of life at the beginning of FOR, no variables were significantly associated with the dependent variable.

**Table 5:** Predictors of survivors’ school/kindergarten related quality of life one year after the end of FOR (*n *= 62)^a^.

	*B*	*SE*	Beta	*T*	*p*
School/kindergarten related quality of life at the beginning of FOR	0.419	0.143	.390	2.939	.005
Time since first measurement time point	−1.662	2.309	−.093	−0.720	.475
Gender rater (reference: female)	−2.513	5.145	−.065	−0.488	.627
Age	−1.097	0.753	−.192	−1.456	.152
Gender (reference: female)	1.560	4.695	.043	0.332	.741
Diagnosis (reference: leukemia)	6.224	5.434	.168	1.145	.257
Physical functioning	−0.049	0.183	−.041	−0.270	.788
Time since end of treatment	0.066	0.110	.080	0.601	.551
Number of siblings	−2.645	2.986	−.115	−0.886	.380
Parental functioning	0.327	0.487	.094	0.672	.504
*R*^2^ (adjusted *R*^2^)	.30 (.16)				
*F*	2.170				
*P*	.035				

^a^ Controlled for the school/kindergarten related quality of life at the beginning of FOR, the time since first measurement time point and the gender of the rater.

## 4 Discussion and conclusions

Similar to findings of other studies (18, 46), most parents in our sample were gainfully employed at the beginning of FOR. However, while most fathers were working full-time, many mothers worked part-time, were homemakers or sought employment. From the time of diagnosis until the time of the survey, many parents reported changes in their professional situation (e.g., reduction of working time, sick leave, unpaid leave, termination). Mothers reported these changes to a greater extent than fathers, possibly due to traditional parental roles. One year later, changes included an increase of working time, job change or return to work. Parental functioning increased significantly from the beginning of the FOR until one year after end of rehabilitation. While caretaking of the ill child may have taken up much energy and time of the parents during active treatment (17), the parental situation with regard to functioning seems to have improved one year after FOR. A leukemia diagnosis in comparison to a CNS tumor diagnosis, a higher functioning at the beginning of FOR and a shorter time span since the end of intensive cancer treatment are associated with higher functioning one year after the end of FOR. Children with CNS tumors may suffer from more severe long-term consequences (47) and, hence, require more support from their parents. Moreover, children with less complications during treatment and course of disease may receive FOR rather immediately after treatment.

Looking at the survivors' school career at the beginning of the FOR, the school enrollment of some children was deferred, and others repeated a class once or multiple times. Parents particularly reported difficulties in the survivors' work pace, concentration, stress resilience and empathy. Our findings indicate differences between children with CNS and leukemia. As these difficulties may be distinct, but still do not reach the threshold for (neuro-)developmental diagnoses, this might interfere with the provision of adequate and necessary treatments and support offers (48). Therefore, the difficulties could be particularly addressed in support offers during aftercare.

The health-related quality of life with regard to school/kindergarten at both measurement time points was lower than in the general population during the study period (49). Besides the control variable school/kindergarten related quality of life at the beginning of FOR, no predictors of the survivor's school/kindergarten related quality of life one year after the end of FOR could be identified. Experiences in school and kindergarten may be influenced by other factors, we did not include into our analyses. As kindergarten and school takes a large part of daily life and social participation, interventions should be implemented, not only to enhance school attendance, but also school experience. Relevant aspects could be the attitude of school staff or collaboration of parents, school staff and healthcare providers (50).

Parents and their children seek for normalcy after the end of cancer treatment. Results indicate, that one year after the end of FOR, most children reintegrate fully in school/kindergarten. However, reintegration is associated with burden, cutbacks and additional support. Parents reported that children returned e.g., only on an hourly basis or they returned hourly until the full reintegration. Some survivors were supported by an integration assistant or received other support (e.g., transport service, school support assistant, compensation of disadvantages at school).

The main limitation of this study is the limited generalizability. We only included families that participated in one specific rehabilitation clinic and we only included leukemia and CNS-tumor survivors and differences in the timespan between diagnosis and entry in the FOR. We did not include a control group over time without any intervention. Another limitation is the operationalization of reintegration. Since the assessment of work and school reintegration is complex, comparisons with other studies might be difficult, e.g., lack of reference values from a general sample for the ULQIE. However, we combined validated questionnaires with self-developed items to describe the reintegration process in order to provide a more comprehensive view of reintegration. This study has also several strengths. We could reach an adequate sample size using a quantitative approach and supplement findings from qualitative studies on reintegration. Moreover, we explicitly included the perspective of both mothers and fathers, while in many other studies on children with cancer, mainly mothers participate. The longitudinal approach enabled us to analyze predictors from the beginning of the FOR allowing for relevant conclusions and practical implications with regard to the reintegration processes after the end of active treatment.

Parents and survivors experience major changes and burdens in their work and school/kindergarten life. Specifically, the mothers' work life is particularly affected. Whereas the parent's work situation seems to improve from the beginning of the FOR until one year after end of FOR (e.g., increased functioning, increased working time), the survivor's quality of life with regard to school/kindergarten decreased slightly, though not significantly, and remained below average during the study period. Parents also report various difficulties in their child's skills that are relevant for a successful reintegration into kindergarten or school. Therefore, psychosocial as well as practical support offers (e.g., integrations assistants) during and after cancer treatment are both highly indicated to alleviate the burden of the families and support them in their struggle to reintegrate into work, school and kindergarten.

The FOR is an aftercare program that supports families with the re-entry into daily life. FOR can provide social medical consultation and initiate relevant support offers for reintegration. Some parents in this study reported, that their children were supported by a transport service, a school support assistant or compensatory measures of disadvantages at school. However, it should be complemented by further targeted support programs in the long term, since difficulties may not dissolve over time. Offering continuous support beginning during treatment (e.g., continuity of education) could reduce long-term problems in the survivor's education and facilitate reintegration after the end of intensive cancer treatment (32).

## Data Availability

The raw data supporting the conclusions of this article will be made available by the authors after consultation with the data protection manager.

## References

[1] Kaatsch P. Epidemiology of childhood cancer. Cancer Treat Rev. (2010) 36(4):277–85. 10.1016/j.ctrv.2010.02.003 20231056

[2] Jörngården A Mattsson E von Essen L. Health-related quality of life, anxiety and depression among adolescents and young adults with cancer: a prospective longitudinal study. Eur J Cancer. (2007) 43(13):1952–8. 10.1016/j.ejca.2007.05.031 17624761

[3] Phipps S Long A Hudson M Rai SN. Symptoms of post-traumatic stress in children with cancer and their parents: effects of informant and time from diagnosis. Pediatr Blood Cancer. (2005) 45(7):952–9. 10.1002/pbc.20373 15806541

[4] Sawyer M Antoniou G Toogood I Rice M Baghurst P. Childhood cancer: a 4-year prospective study of the psychological adjustment of children and parents. J Pediatr Hematol Oncol. (2000) 22(3):214–20. 10.1097/00043426-200005000-00006 10864052

[5] Ljungman L Cernvall M Grönqvist H Ljótsson B Ljungman G von Essen L. Long-term positive and negative psychological late effects for parents of childhood cancer survivors: a systematic review. PLoS One. (2014) 9(7):e103340. 10.1371/journal.pone.0103340 25058607 PMC4110004

[6] Wakefield CE McLoone JK Butow P Lenthen K Cohn RJ. Parental adjustment to the completion of their child’s cancer treatment. Pediatr Blood Cancer. (2011) 56(4):524–31. 10.1002/pbc.22725 21298736

[7] Wenninger W Helmes A Bengel J Lauten M Voelkel S Niemeyer CM. Coping in long-term survivors of childhood cancer: relations to psychological distress. Psychooncology. (2013) 22(4):854–61. 10.1002/pon.3073 22461240

[8] Schrag NM McKeown RE Jackson KL Cuffe SP Neuberg RW. Stress-related mental disorders in childhood cancer survivors. Pediatr Blood Cancer. (2008) 50(1):98–103. 10.1002/pbc.21285 17610265

[9] Reinfjell T Lofstad GE Nordahl HM Vikan A Diseth TH. Children in remission from acute lymphoblastic leukaemia: mental health, psychosocial adjustment and parental functioning. Eur J Cancer Care. (2009) 18(4):364–70. 10.1111/j.1365-2354.2008.00954.x 19473372

[10] Engelen V Koopman HM Detmar SB Raat H van de Wetering MD Brons P Health-related quality of life after completion of successful treatment for childhood cancer. Pediatr Blood Cancer. (2011) 56(4):646–53. 10.1002/pbc.22795 21298753

[11] Labay LE Mayans S Harris MB. Integrating the child into home and community following the completion of cancer treatment. J Pediatr Oncol Nurs. (2004) 21(3):165–9. 10.1177/1043454204264396 15296047

[12] Kazak AE Alderfer M Rourke MT Simms S Streisand R Grossman JR. Posttraumatic stress disorder (PTSD) and posttraumatic stress symptoms (PTSS) in families of adolescent childhood cancer survivors. J Pediatr Psychol. (2004) 29(3):211–9. 10.1093/jpepsy/jsh022 15131138

[13] McCaffrey CN. Major stressors and their effects on the well-being of children with cancer. J Pediatr Nurs. (2006) 21(1):59–66. 10.1016/j.pedn.2005.07.003 16428015

[14] Barrera M Shaw AK Speechley KN Maunsell E Pogany L. Educational and social late effects of childhood cancer and related clinical, personal, and familial characteristics. Cancer. (2005) 104(8):1751–60. 10.1002/cncr.21390 16130127

[15] Soanes L Hargrave D Smith L Gibson F. What are the experiences of the child with a brain tumour and their parents? Eur J Oncol Nurs. (2009) 13(4):255–61. 10.1016/j.ejon.2009.03.009 19423391

[16] Sloper P. Needs and responses of parents following the diagnosis of childhood cancer. Child: Care. Health Dev. (1996) 22(3):187–202. 10.1046/j.1365-2214.1996.788788.x 8735673

[17] Roser K Erdmann F Michel G Winther JF Mader L. The impact of childhood cancer on parents’ socio-economic situation—a systematic review. Psychooncology. (2019) 28(6):1207–26. 10.1002/pon.5088 30970149

[18] Lindahl Norberg A Montgomery SM Bottai M Heyman M Hovén EI. Short-term and long-term effects of childhood cancer on income from employment and employment status: a national cohort study in Sweden. Cancer. (2017) 123(7):1238–48. 10.1002/cncr.30436 27870013

[19] Lindahl Norberg A Steneby S. Experiences of parents of children surviving brain tumour: a happy ending and a rough beginning. Eur J Cancer Care. (2009) 18(4):371–80. 10.1111/j.1365-2354.2008.00976.x 19490006

[20] Wakefield CE McLoone JK Evans NT Ellis SJ Cohn RJ. It’s more than dollars and cents: the impact of childhood cancer on parents’ occupational and financial health. J Psychosoc Oncol. (2014) 32(5):602–21. 10.1080/07347332.2014.936653 24988134

[21] Mader L Rueegg CS Vetsch J Rischewski J Ansari M Kuehni CE Employment situation of parents of long-term childhood cancer survivors. PLoS One. (2016) 11(3):e0151966. 10.1371/journal.pone.0151966 26990301 PMC4798766

[22] Schröder HM Lilienthal S Schreiber-Gollwitzer BM Grießmeier B Hesselbarth B Lein-Köhler I Psychosoziale Versorgung in Der Pädiatrischen Onkologie und Hämatologie (2019). Available online at: https://www.awmf.org/leitlinien/detail/ll/025-002.html (accessed October 2020).

[23] Arbeitsgemeinschaft Familienorientierte Rehabilitation. Positionspapier Zur Familienorientierten Rehabilitation Bei Krebskranken Kindern (2001). Available online at: https://www.kinderkrebsinfo.de/fachinformationen/psapoh/rehabilitation/index_ger.html (accessed April 2017).

[24] Krauth KA. Family-oriented rehabilitation (FOR) and rehabilitation of adolescents and young adults (AYA) in pediatric oncology. Oncol Res Treat. (2017) 40(12):752–8. 10.1159/000484609 29151110

[25] Bauer CP Petermann F. DGRW-update: rehabilitation bei kindern und jugendlichen [DGRW-update: medical rehabilitation with children and adolescents]. Rehabilitation (Bonn). (2010) 49(4):217–23. 10.1055/s-0030-1261904 20677117

[26] Peikert ML Inhestern L Bergelt C. The role of rehabilitation measures in reintegration of children with brain tumours or leukaemia and their families after completion of cancer treatment: a study protocol. BMJ Open. (2017) 7:e014505. 10.1136/bmjopen-2016-014505 PMC5724106 28801389

[27] Peikert ML Inhestern L Krauth KA Escherich G Rutkowski S Kandels D Fear of progression in parents of childhood cancer survivors: prevalence and associated factors. J Cancer Surv. (2022) 16:823–33. 10.1007/s11764-021-01076-w PMC9300493 34302272

[28] Peikert ML Inhestern L Krauth KA Escherich G Rutkowski S Kandels D Fear of progression in parents of childhood cancer survivors: a dyadic data analysis. Psycho-Oncology. (2020) 29(10):1678–85. 10.1002/pon.5508 32779255

[29] Winzig J Inhestern L Paul V Nasse ML Krauth KA Kandels D Parent-reported health-related quality of life in pediatric childhood cancer survivors and factors associated with poor health-related quality of life in aftercare. Qual Life Res. (2023) 32:2965–74. 10.1007/s11136-023-03436-8 37204653 PMC10474174

[30] Paul V Inhestern L Winzig J Nasse ML Krauth KA Rutkowski S Emotional and behavioral problems of pediatric cancer survivors and their siblings: concordance of child self-report and parent proxy-report. Psycho-Oncology. (2023) 32(8):1248–56. 10.1002/pon.6175 37303105

[31] Peikert ML Inhestern L Krauth KA Escherich G Rutkowski S Kandels D Returning to daily life: a qualitative interview study on parents of childhood cancer survivors in Germany. BMJ Open. (2020) 10(3):e033730. 10.1136/bmjopen-2019-033730 PMC7064139 32152163

[32] Inhestern L Peikert ML Krauth KA Escherich G Rutkowski S Kandels D Parents’ perception of their children’s process of reintegration after childhood cancer treatment. PloS One. (2020) 15(10):e0239967. 10.1371/journal.pone.0239967 33002084 PMC7529258

[33] Lang TA Altman DG. Basic statistical reporting for articles published in biomedical journals: the “statistical analyses and methods in the published literature” or the SAMPL guidelines. Int J Nurs Stud. (2015) 52(1):5–9. 10.1016/j.ijnurstu.2014.09.006 25441757

[34] Kaatsch P Grabow D Spix C. German Childhood Cancer Registry—Annual Report 2018 (1980–2017). Mainz: Institute of Medical Biostatistics, Epidemiology and Informatics (IMBEI) at the University Medical Center of the Johannes Gutenberg University Mainz (2019).

[35] Goldbeck L Storck M M. Das ulmer lebensqualitäts-inventar für eltern chronisch kranker kinder (ULQIE) [ULQIE: a quality-of-life inventory for parents of chronically ill children]. Z Klin Psychol Psychother. (2002) 31(1):31–9. 10.1026/0084-5345.31.1.31

[36] Spitzer RL Kroenke K Williams JB Löwe B. A brief measure for assessing generalized anxiety disorder: the GAD-7. Arch Intern Med. (2006) 166(10):1092–7. 10.1001/archinte.166.10.1092 16717171

[37] American Psychiatric Association. Diagnostic and Statistical Manual of Mental Disorders: DSM-IV-TR. 4th ed Washington, DC: American Psychiatric Association (2000).

[38] Löwe B Decker O Müller S Brähler E Schellberg D Herzog W Validation and standardization of the generalized anxiety disorder screener (GAD-7) in the general population. Med Care. (2008) 46(3):266–74. 10.1097/MLR.0b013e318160d093 18388841

[39] Löwe B Kroenke K Herzog W Gräfe K. Measuring depression outcome with a brief self-report instrument: sensitivity to change of the patient health questionnaire (PHQ-9). J Affect Disord. (2004) 81(1):61–6. 10.1016/S0165-0327(03)00198-8 15183601

[40] Kroenke K Spitzer RL Williams JB. The PHQ-9: validity of a brief depression severity measure. J Gen Intern Med. (2001) 16(9):606–13. 10.1046/j.1525-1497.2001.016009606.x 11556941 PMC1495268

[41] Martin A Rief W Klaiberg A Braehler E. Validity of the brief patient health questionnaire mood scale (PHQ-9) in the general population. Gen Hosp Psychiatry. (2006) 28(1):71–7. 10.1016/j.genhosppsych.2005.07.003 16377369

[42] Ravens-Sieberer U Bullinger M. Der kindl-R fragebogen zur erfassung der gesundheitsbezogenen lebensqualität bei kindern und jugendlichen—revidierte form. In: Schumacher J Klaiberg A Brähler E, editors. Diagnostische Verfahren Zu Lebensqualität und Wohlbefinden. Göttingen: Hogrefe (2003). p. 184–8.

[43] Ravens-Sieberer U Bullinger M. Questionnaire for Measuring Health-Related Quality of Life in Children and Adolescents. Revised version. Manual (2000). Available online at: https://www.kindl.org/deutsch/manual/ (accessed September 2020).

[44] Erhart M Ellert U Kurth BM Ravens-Sieberer U. Measuring adolescents’ HRQoL via self reports and parent proxy reports: an evaluation of the psychometric properties of both versions of the KINDL-R instrument. Health Qual Life Outcomes. (2009) 7(1):77. 10.1186/1477-7525-7-77 19709410 PMC2749015

[45] Ellert U Ravens-Sieberer U Erhart M Kurth BM. Determinants of agreement between self-reported and parent-assessed quality of life for children in Germany—results of the German health interview and examination survey for children and adolescents (KiGGS). Health Qual Life Outcomes. (2011) 9:102. 10.1186/1477-7525-9-102 22111939 PMC3286376

[46] Wikman A Hovén E Cernvall M Ljungman G Ljungman L von Essen L. Parents of children diagnosed with cancer: work situation and sick leave, a five-year post end-of-treatment or a child’s death follow-up study. Acta Oncol (Madr). (2016) 55:1152–7. 10.3109/0284186X.2016.1167956 27159219

[47] Mertens AC Brand S Ness KK Li Z Mitby PA Riley A Health and well-being in adolescent survivors of early childhood cancer: a report from the childhood cancer survivor study. Psychooncology. (2014) 23:266–75. 10.1002/pon.3414 24123762 PMC3988531

[48] Oswald K Bitensky D Stuchell E Edmonds A Richard A Hodges E Neuropsychological assessment in pediatric oncology survivorship care: utilization of services, results of evaluation, and educational and behavioral health outcomes. Support Care Cancer. (2021) 29:7965–74. 10.1007/s00520-021-06401-8 34213645

[49] Ravens-Sieberer U Ellert U Erhart M. Gesundheitsbezogene lebensqualität von kindern und jugendlichen in deutschland. Bundesgesundheitsblatt. (2007) 50:810–8. 10.1007/s00103-007-0244-4 17514467

[50] Vanclooster S Van Hoeck K Peremans L Bilsen J Van Der Werff Ten Bosch J Laureys G Reintegration into school of childhood brain tumor survivors: a qualitative study using the international classification of functioning, disability and health—children and youth framework. Disabil Rehabil. (2021) 43(18):2610–20. 10.1080/09638288.2019.1708484 31910686

