# Supplementary material for: Reintegration into school, kindergarten and work in families of childhood cancer survivors after a family-oriented rehabilitation program
Source: Front Pediatr. 2024 Mar 7;12:1288567. doi: 10.3389/fped.2024.1288567 (PMC10954838; doi:10.3389/fped.2024.1288567)
Supplement: Supplementary file 1 [file Table1.pdf]

S1 Table. Comparison of ICF ratings of parents that only participated at the beginning of FOR and the ratings of parents that participated at both measurement timepoints

Item		Drop Out		
		No	Yes	p
solving tasks that require capacity of memory	Never	2	4	.884
	Rarely	5	4	
	Sometimes	17	19	
	Often	44	39	
	Always	18	17	
performing actions in an adequate work pace	Never	1	2	.736
	Rarely	10	15	
	Sometimes	28	23	
	Often	37	33	
	Always	7	7	
concentrating	Never	-	-	-[a]
	Rarely	8	13	
	Sometimes	28	37	
	Often	37	25	
	Always	13	7	
Having energy for school/kindergarten	Never	1	1	.534
	Rarely	6	5	
	Sometimes	17	25	
	Often	37	33	
	Always	21	14	
Understanding and solving tasks	Never	-	2	-[a]
	Rarely	3	8	
	Sometimes	22	21	
	Often	44	42	
	Always	17	10	
Listening or observing attentively	Never	-	1	-[a]
	Rarely	7	9	
	Sometimes	15	24	
	Often	42	32	
	Always	21	15	
withstanding stress	Never	4	6	.318
	Rarely	20	28	
	Sometimes	26	26	
	Often	30	18	
	Always	4	4	
understanding feelings and thoughts of others	Never	2	2	.889
	Rarely	10	14	

Sometimes	21	19
Often	36	33
Always	15	12

[a] no significance test due to small number in cells
