# Supplementary material for: Reintegration into school, kindergarten and work in families of childhood cancer survivors after a family-oriented rehabilitation program
Source: Front Pediatr. 2024 Mar 7;12:1288567. doi: 10.3389/fped.2024.1288567 (PMC10954838; doi:10.3389/fped.2024.1288567)
Supplement: Supplementary file 2 [file Table2.pdf]

S2 Table. Comparison of school subscale of the KINDL in children with CNS and Leukemia before and one year after FOR

		N	M	SD	p
KINDL school subscale before FOR	CNS	49	70,7	15,8	.086
	Leukemia	79	75,9	17,2	
KINDL school subscale one year after FOR	CNS	31	69,8	17,7	.329
	Leukemia	51	74,0	19,6	

S3 Table. Comparison of ICF related skills in children with CNS and Leukemia before FOR

Item		CNS	Leukemia	p
solving tasks that require capacity of memory	Never	2	4	.012
	Rarely	7	2	
	Sometimes	17	19	
	Often	28	55	
	Always	7	28	
performing actions in an adequate work pace	Never	2	1	.006
	Rarely	15	10	
	Sometimes	20	31	
	Often	20	50	
	Always	1	13	
concentrating	Never	-	-	-[a]
	Rarely	7	14	
	Sometimes	28	37	
	Often	20	42	
	Always	5	15	
Having energy for school/kindergarten	Never	2	0	.013
	Rarely	7	4	
	Sometimes	15	27	
	Often	26	44	
	Always	6	29	
Understanding and solving tasks	Never	1	1	.101
	Rarely	6	5	
	Sometimes	18	25	
	Often	32	54	
	Always	4	23	
Listening or observing attentively	Never	1	0	-[a]
	Rarely	4	12	
	Sometimes	14	25	
	Often	32	42	
	Always	9	27	
withstanding stress	Never	4	6	.221
	Rarely	14	34	
	Sometimes	24	28	
	Often	13	35	
	Always	4	4	
understanding feelings and thoughts of others	Never	0	4	-[a]
	Rarely	10	14	
	Sometimes	17	23	
	Often	24	45	
	Always	7	20	

[a] no significance test due to small number in cells

S4 Table. Comparison of ICF related skills in children with CNS and Leukemia one year after FOR

Item		CNS	Leukemia	p
solving tasks that require capacity of memory	Never	3	0	-[a]
	Rarely	2	4	
	Sometimes	5	10	
	Often	17	24	
	Always	6	18	
performing actions in an adequate work pace	Never	2	1	.062
	Rarely	10	5	
	Sometimes	9	19	
	Often	10	23	
	Always	2	8	
concentrating	Never	1	0	-[a]
	Rarely	6	6	
	Sometimes	13	16	
	Often	9	29	
	Always	4	5	
Having energy for school/kindergarten	Never	1	1	.258
	Rarely	2	4	
	Sometimes	10	7	
	Often	15	28	
	Always	5	16	
Understanding and solving tasks	Never	1	0	-[a]
	Rarely	2	0	
	Sometimes	11	12	
	Often	12	31	
	Always	7	13	
Listening or observing attentively	Never	1	0	-[a]
	Rarely	1	3	
	Sometimes	8	15	
	Often	17	22	
	Always	6	16	
withstanding stress	Never	1	0	-[a]
	Rarely	13	12	
	Sometimes	11	24	
	Often	7	17	
	Always	1	3	
understanding feelings and thoughts of others	Never	1	0	-[a]
	Rarely	3	4	
	Sometimes	10	15	
	Often	11	24	
	Always	8	13	

[a] no significance test due to small number in cells
